# On an Imperfectly Known Function of the Pancreas, Namely Digestion of Nitrogenous Food, with Comparative Experiments on Gastric and Intestinal Digestion, Followed by a Few Clinical Deductions

**Published:** 1859-07

**Authors:** L. Corvisart


### ON AN IMPERFECTLY KNOWN FUNCTION OE THE PANCREAS, NAMELY DIGESTION OF NITROGENOUS FOOD, WITH COMPARATIVE EXPERIMENTS ON GASTRIC AND INTESTINAL DIGESTION, FOLLOWED BY A FEW CLINICAL DEDUCTIONS.

By L. Corvisart, M.D., *London Lancet.*

[The following important propositions, deduced from Dr.

Corvisart's skillfully conducted experiments, were sent to us, in manuscript, by the author, some time since, and we regret that very great pressure of matter prevented these valuable contributions to science being inserted before.—*Ed. L.*]

*General Propositions, forming the Summary of an Essay, published with the above title, and read before the Academy of Medicine of Paris; the first part in* 1857, *and the second in February*, 1858.

Very little is known about the manner in which the animal or nitrogenous food is digested in the *bowel;* and science has not advanced one step since the discovery of Purkinje and Pappenheim (1836) respecting the dissolving action which the pancreatic juice may exercise on such food—a discovery which has indeed remained almost unnoticed.

The physiological and experimental investigations on the *second digestion* (intestinal digestion), of which I have given an account (in the before-mentioned essay), have led to the follow- important results:—These are twofold: One group, of a physiological and direct character, are deduced from actual experiments. The other, of a pathological and indirect nature, are deductions or corollaries, which, as it seems to me, throw some light on clinical medicine.

### I.—*Physiological Propositions.*

1. Nitrogenous food is digested both by the stomach and the pancreas.

2. The pancreas is, as it were, a supplementary organ, whose action, after copious meals, is added to that of the stomach.

3. Both digestions are of the same nature, as any article of food subjected to either is transformed into the self-same nutrimentive product (albuminose or peptone).

4. The pancreatic juice has peculiar reactions under the influence of heat or certain agents, which reaction *the gastric juice does not present.* As this difference in the juices is found when they are both charged with peptones, after digestion, it has erroneously been supposed that the peptones also differed. This pardonable error, being pointed out, will hardly again be fallen into.

5. When an article of nitrogenous food, or a portion of it, has undergone a thorough gastric digestion, the pancreatic juice no longer acts upon it, and does not transform it into another peptone.

6. The pancreatic juice is intended to act upon that part of albuminoid substances which has left the stomach before being transformed into albuminose.

7. The amount of action of the pancreas may, in certain cases, be equal to that of the stomach.

8. If the mere quantity of secreted fluid were alone taken into account, the stomach might be looked upon as the more powerful, for the gastric juice is ten times more abundant than the pancreatic juice; but the latter is, to make up the difference, ten times richer in ferment (pancreatine).

9. The gastric juice has the advantage of a prolonged contact and stirring with the food; but the pancreatic juice has, on the other hand, the faculty of acting upon azotized aliments equally well, either in an alkaline, neutral, or acid state; it also acts three times quicker than the gastric juice.

10. Every thing is so disposed in the duodenum, that the pancreatic juice acts immediately it comes in contact with the food; and every thing is so arranged in the stomach that a large part of the food is transformed into peptone, the remaining part being, at the very least, so prepared, as rapidly to undergo the pancreatic digestion.

11. This preparation, which varies according to the quality and quantity either of the food or the gastric juice, etc., consists sometimes in a simple imbibition, sometimes in a dissevering or an extreme division, and sometimes in a solution. Pancreatic digestion, being forcibly very rapid, is usefully assisted by this preparation, the stomach acting respecting the pancreas in the same manner as the teeth do respecting gastric digestion.

12. It is, however, to be noticed that the pancreatic juice is able to accomplish, unassisted, the digestion of food which has not been subjected to that gastric preparation or division, in the same way as the gastric juice can digest food without extraneous help. Hence, pieces of albuminoid substances, being *directly* placed into the intestine in a raw state—that is to say, without any preparation—are perfectly and completely digested, the process being, however, somewhat slow. The pancreatic juice can, by its own unassisted energy, carry on the digestion of nitrogenous food, without requiring the adjunction either of the intestinal juice or the bile, to gain digestive properties. The digestion of azotized food, performed in glass jars over the water bath, by means of the pancreatic juice or isolated pancreatine, goes on in the same manner as in the duodenum.

13. When the gastric and pancreatic juices are separated, and act in succession, each performs its function completely, and the quantity of albuminose produced may thus be doubled.

14. But it is a remarkable fact, that when these two digestive ferments meet in a state of purity, the two digestions are no longer freely carried on. The mixture, far from doubling the produce, may reduce it to naught, for pepsine and pancreatine destroy each other under these non-physiological circumstances.

15. Nature, in the normal state, prevents this conflict, by three distinct means—1stly, by the pylorus, which separates the two ferments; 2ndly, by the very gastric digestion through which pepsine exhausts and abolishes itself in the formation of peptone; 3dly, by the bile, which destroys the activity of the gastric ferment, as has been shown by Pappenheim.

16. Bile does not precipitate the peptone produced by the influence of the stomach so as to destroy digestion and necessitate its being again begun. On the contrary, the bile itself is precipitated by the acid of the gastric juice or of the chyme.

17. The nature of the nitrogenous food has much to do with the quantity of peptone which the two successive digestions can produce for the requirements of economy. I have thus found in my experiments, that whilst musculine and caseine yielded almost one ounce of perfect peptone, albumen, or gelatigenous textures, though given in the same quantity, yielded hardly half an ounce.

18. At the outset, gastric or pancreatic digestion destroys the most characteristic properties of the various albuminoid substances. It liquefies insoluble ones, deprives albumen of its coagulability, and caseine of its property of coagulating by rennet. It also deprives gelatine of its property of turning into jelly, and musculine of being precipitated by chloride of sodium, etc. In short, it transforms all the substances into albuminose and peptone.

The different kinds of albuminose, although their individual reactions are much less marked than those of the albuminoid substances whence they are derived, have, nevertheless, distinct characters.

19. The nature of peptones varies as the nitrogenous substances from which they are derived. This variety satisfies the different (plastic?) requirements of the economy.

20. The peptones which are most alike and most difficult to distinguish from each other, are, the albumen-peptone; just as if the articles of food from which these peptones are derived were less different from each other than is generally supposed.

Fibrine-peptone and caseine-peptone are more easily distinguished from each other, and from the substances above named. From the slight differences existing between azotized articles of food, or peptones, there arises a kind of unstable equilibrium, favorable to the work of assimilation performed by the tissues of the body.

21. The generic character of peptones is, that they are always soluble in water, be the latter acid, neutral, or alkaline, which circumstance secures an easy circulation in the organism. Heat does not coagulate peptones, and hardly any of them are precipitated by acetate of lead. Besides, they resist insoluble metallic combinations a great deal better than nitrogenous articles of food.

22. Peptones form a genus, as well defined as the albuminoid genus. It is, however, evident, that by the progress of science, their nature will eventually be more exactly determined than can be done at the present period.

23. Some physiologists persist in the erroneous belief that the stomach merely swells or divides the food without dissolving it. How can they, however, withstand the testimony of the scales, which plainly show that, even where the weight of the food is considerable, every albuminoid article of food subjected to the action of the stomach is not merely divided, but dissolved, passes through the filter, and is absorbed by the membranes!

24. Others have maintained that the gastric juice, acting on nitrogenous food, produces only gelatine. They, however, lose sight of the fact, that the characters which place gelatine in a peculiar albuminoid class, have never been discovered in the chyme after a digestion of fibrine, caseine, musculine, or albumine, even when the chyme was neutralized; and that, moreover, gelatine itself completely loses its specific characters, in consequence of undergoing digestion in the gastric juice.

25. Others, finally, resting on the hypothesis, that the albumen of the blood is nothing but the digested matters themselves, maintain, that the pepetones are reduced to albumen, by losing their acidity—viz: by being neutralized. Such an error can hardly exist, except albumen and fibrine be alone taken into account, excluding all other aliments; as an incomplete digestion of the albumen and fibrine may lead to confusion. Crude albumen, in fact, always partly escapes gastric digestion; ill-digested fibrine is transformed into albumen only (caseiform); these two cases excepted, if experiments be made on the produce of concrete and washed albumen, of caseine, musculine, or gelatine, regularly digested by the stomach, no doubt can any longer be entertained. These gastric peptones never contain any albumen.

26. The peptones, either received or produced by the pancreatic juice, do not, any more than the latter, form any new albumen, and, whether they be primarily or consecutively acid, alkaline or neutral, do not increase by an appreciable weight the coaguble albumen which the pancreatic juice, pure and without peptone, *normally contains.*

27. During the three hours which follow a meal (when digestive solution, transformation, and absorption are not much advanced), the blood of the vena portæ (compared to the venous blood generally) does not become charged with a noticeable quantity of nitrogenous matter through digestive absorption; whilst on the other hand, the elements of the blood, globules and fibrine become changed into albumen (caseiform) by a commencement of digestion, either in the intestine or the water bath, under the influence of the alkaline pancreatic juice.

28. Now, if it be considered that, during the first three hours of digestion—1stly, the pancreatic juice poured into the duodenum remains therein in a pure and active state; 2ndly, that this juice can pass into the vena portæ (for absorption by the mesenteric veins is not suspended); 3rdly, that this same juice can act in such an alkaline medium as the blood. If, moreover, it be considered that during those very three hours, a large portion of the globules and fibrine of the blood of the vena portæ is, weights remaining equal, transformed in that vein into albumen (which is a commencement of transformation similar to that which they would have undergone in the intestine under the influence of this same pancreatic juice) we can hardly refuse our assent to the hypothesis of *a true intra-venous digestion*, which hypothesis I confidently put forward.

29. No actually differential character has ever been pointed out between the nitrogenous matters which go by the name of extractive, and the albuminose, which is generated by gastric or pancreatic digestion. Now, it should be noticed that the lacteals, *the vena portæ, and the hepatic veins which are its continuation*, or in other words, the vessels which most directly receive the product of digestion,—are by far richer in extractive matter (albuminose) than the rest of the blood. It may, moreover, be noted that they are also richer in glucose.

30. The nutritive richness of the hepatic vessels (albuminose and glucose being contained in them) may be explained by the gastro-intestinal absorption, to which is energetically added prolonged intra-venous digestion, although the liver has no share in the process.

II.—*Corollaries, vel Pathological Deductions.*

A. We may take it as almost certain that there exists (as regards albuminoid aliments) a duodenal dyspepsia, caused by the vitiation, insufficiency, or absence of the pancreatic juice, the symptoms of which appear only from the second or third hour of digestion, with a deeper-seated pain than is felt in gastric dyspepsia. (See Propositions 1, 2, 3, 6, 7.) The internal use of pancreatine is indicated* in cases of pancreatic duodenal dyspepsia.

B. Secondary duodenal dyspepsia may be the result of an almost total absence of that kind of division which food, under the least favorable circumstances, undergoes by means of the gastric juice before that food has been transformed into peptone. Pancreatic digestion is then slower, just as gastric digestion is slower when the teeth have not duly performed their functions. This secondary pancreatic dyspepsia may be cured by the treatment suited to the primary gastric dyspepsia.

C. Another secondary duodenal dyspepsia may arise, either from an excess of gastric juice, or from a patency of the pylorus; for in these two individual cases the gastric juice reaches the duodenum in unfortunately retaining all its active properties, which latter are prejudicial to the action of the pancreatic juice. (See Propositions 13, 14, 15 and 16.)

D. A third duodenal dyspepsia may arise from deficient biliary secretion, this deficiency being followed by the same unpleasant effects as are noticed in the two preceding cases, on account of the non-destruction of the activity of the gastric juice in the duodenum.

E. A peculiar kind of dyspepsia, which might be called of the portal vein, or hepatic, may arise from the vitiation of the intra-venous digestion.

F. Certain symptoms of dyspepsia, gastralgia, enteralgia, or

---

* Last year Dr. Corvisart made some clinical experiments on the therapeutic use of pure pancreatine. The difficulties he met with are recorded in the Gazette Hebdomadaire of Paris, May, 1857, p. 321, 322. Dr. G. Harley, who read a paper on digestion (just twelve months after the above date) at the meeting of the British Association for the Advancement of Science, seems never to have heard of Dr. Corvisart's article on the subject. Dr. Harley maintains, in opposition to the latter physician's statements, that in the administration of duodenal ferment, it is not necessary to imitate nature, who prevents pancreatine from passing into the stomach. For the causes of the difficulties met with by Dr. Corvisart, and the means to overcome them, see Propositions 13, 14 and 15, paragraphs C and D of the summary, and page 51 of the Essay.

hepetalgia, may erroneously be attributed to the stomach, the intestine, or the liver; these symptoms may simply be the result of the absorption of a too abundant, too active, or too irritating pancreatic juice by the vena portæ.

G. Bile, when it reaches the stomach, destroys the activity of the gastric juice within that organ, whether it penetrates the cavity pathologically through the pylorus or by the mouth and cardia. The knowledge of this fact may lead to the employment of bile to counteract the morbid super-abundance of the gastric juice.

H. The economy is supplied with a variable weight of peptone, though the weight of different kinds of nitrogenous articles of food and digestive force remain the same, the weight of the peptones varying according to the kind of nitrogenous food. It is a great error in hygienics to esteem the trophic, or nourishing power of a nitrogenous article of food, simply by the amount of nitrogen it contains. The trophic, or alimentary standard of food, is not so easily fixed.

I. When it is more urgent to allay pain and irritation about the digestive organs than to restore muscular energy, the food should consist of that kind of aliment which is most quickly and completely dissolved, whatever be the amount of peptone it yields.

J. But when it is more important rapidly to restore muscular force than to allay gastro-intestinal pain, we should, on the contrary, give such food which, the digestive force being the same, yields the greatest weight of peptone, though that food be likely to dissolve and digest slowly. (See Proposition 17.)

K. He who digests with one organ only (stomach or pancreas), is thereby put on half allowance as regards peptone; and he who eats only albumen or gelatinous tissue (instead of caseine or musculine, which yields double as much peptone), is also put upon half allowance; and with a normal and equal digestive force, is only half nourished. (See Proposition 17.)

In the two preceding cases, an over-activity either of the one organ (first case), or of both organs (second case), may occur, and extract from the food the full allowance of peptone. But we must not long trust this extreme functional exertion; for any persisting over-activity must sooner or later end in exhaustion.

L. We should not give for a long time one kind only of nitrogenous food, not only because one kind of azotized aliment is not capable of repairing the waste of the organism, but also because the same article of food exclusively and continuously (for a week for instance), no longer excites gastric secretion, and no longer fully undergoes the digestive transformation.

M. Most of the peptones upon which I have made experiments, have the peculiarity of not being precipitated by neutral acetate of lead. Now, in all cases where the albuminoid matters of the urine happen to be of the albuminose kind, they remain in solution, in spite of the acetate of lead used to precipitate them. They therefore mask the sugar more effectually than all other ingredients of the urine, when the potash and copper test is employed. The presence of sugar may thus be overlooked when it really exists in the urine.

